# Bilateral Multiligamentous Knee Injuries: A Case Report and Technique Review

**DOI:** 10.1155/2018/3460153

**Published:** 2018-06-19

**Authors:** Malynda S. Messer, Brendan Southam, Brian M. Grawe

**Affiliations:** Department of Orthopaedic Surgery, University of Cincinnati Medical Center, 231 Albert Sabin Way, P.O. Box 670212, Cincinnati, OH 45267-0212, USA

## Abstract

Bilateral knee dislocations are rare musculoskeletal injuries. We report a case of a patient who sustained traumatic bilateral knee dislocations resulting in multiligamentous injuries to both knees. The patient subsequently underwent acute ligamentous reconstructions of both knees performed at 2 weeks and 3 weeks after the initial injury. One year after these procedures, the patient has achieved excellent functional outcomes and has returned to recreational sports.

## 1. Introduction

Multiligamentous knee injuries (MLI) occur as a result of both high- and low-energy traumas to the knee, most commonly due to motor vehicle accidents and sport-related injuries, respectively [1]. Ultra-low-velocity mechanisms have also been observed in obese patients that experience ground-level falls [2–4]. Knee dislocation, which is the primary cause of MLI, is an uncommon orthopaedic injury, only accounting for 0.02% of musculoskeletal trauma [5–7]. However, when such injuries are present, both vascular compromise and neurologic compromise can occur and may potentially threaten limb integrity. A MLI is typically defined as a disruption of at least two of the four major stabilizing ligaments of the knee [7].

Much of the existing literature on MLI has focused on the evaluation and treatment of isolated, unilateral knee injuries. Bilateral MLI are very rare, with most of the literature limited to case reports [8–11]. A recent retrospective case-control study comparing unilateral and bilateral MLI demonstrated a higher rate of concomitant injuries, as well as postoperative complications, in patients with bilateral knee injuries [11]. Furthermore, these patients represent a unique challenge to the surgeon who must evaluate and address numerous ligamentous, meniscal, and bony injuries at the time of reconstruction to effectively restore stability and improve functional outcomes.

This article details a patient who sustained bilateral knee dislocations resulting in MLI. The acute management, surgical reconstruction, and postoperative rehabilitation of this patient are described. Given the uncommon nature of these injuries and the relative paucity of literature regarding their management, increased emphasis has been given to considerations regarding the surgical reconstruction and perioperative management of the patient in this case. The authors obtained the patient's informed written consent for print and electronic publication of this case report.

## 2. Case Report

This case describes a 23-year-old male who was struck by a motor vehicle. Upon arrival at our hospital, the patient had a GCS of 8. FAST exam, chest radiograph, and computed topography (CT) of the head and cervical spine were obtained and were negative.

**Figure 1:**
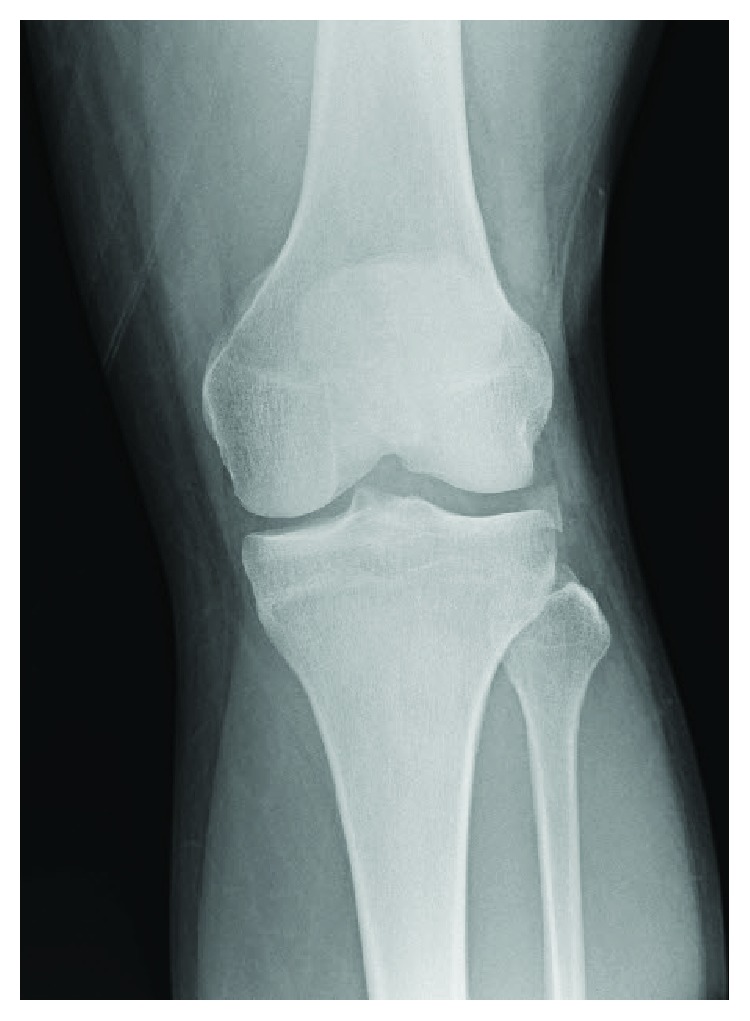
An anteroposterior radiograph of the patient's left knee demonstrating a Segond fracture.

Exam of the lower extremities revealed abrasions over the left knee and tenderness over the lateral joint line with an effusion. The right knee was diffusely tender to palpation without effusion. The patient had palpable pulses in both feet with well-perfused extremities. Ankle brachial indices were performed and found to be >0.9. He demonstrated guarding and pain with the attempted Lachman maneuver of the left knee and slight opening of the left knee joint with varus stress. Radiographs were obtained and revealed a left knee Segond fracture (Figure 1).

**Figure 2:**
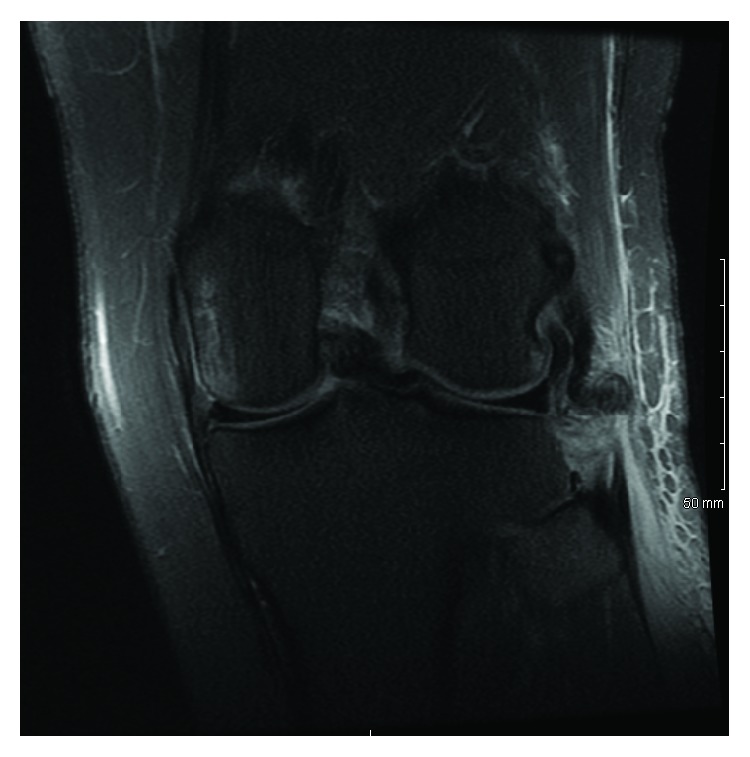
A T2 coronal MRI of the patient's left knee demonstrating a grade III lateral collateral ligament tear with retraction.

**Figure 3:**
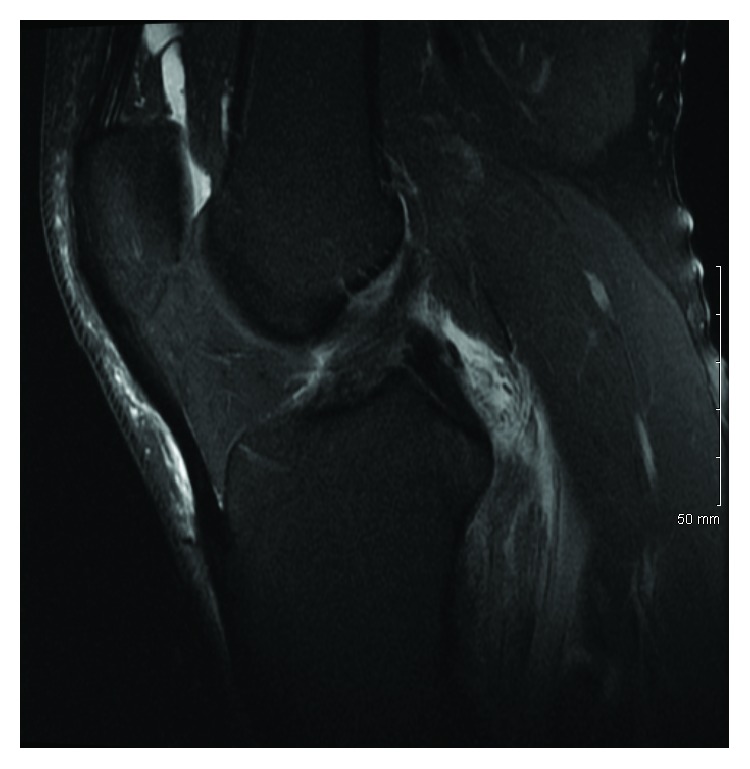
A T2 sagittal MRI of the patient's left knee demonstrating a grade II partial thickness tear of the anterior cruciate ligament.

**Figure 4:**
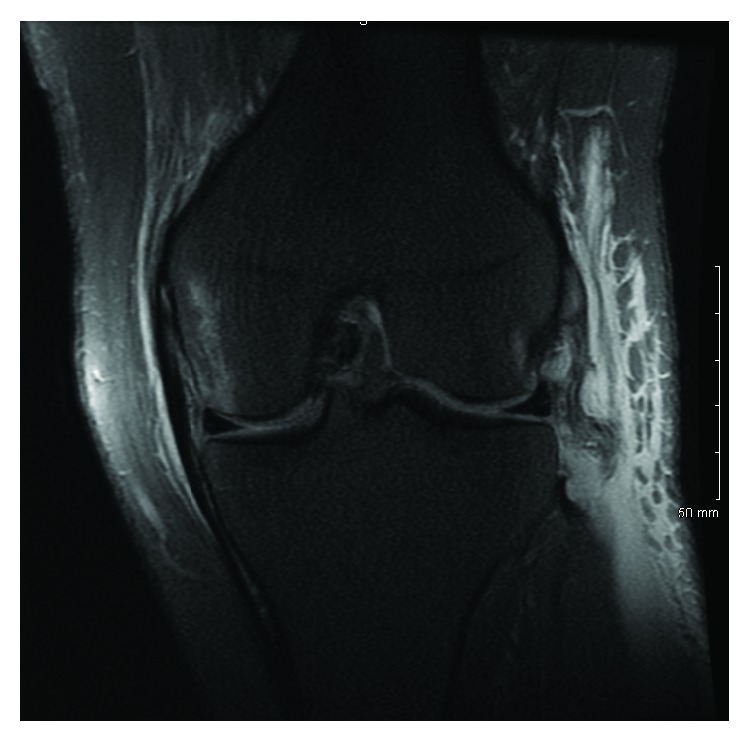
A T2 coronal MRI of the patient's left knee showing a grade I MCL injury with associated subchondral edema in the medial condyle.

**Figure 5:**
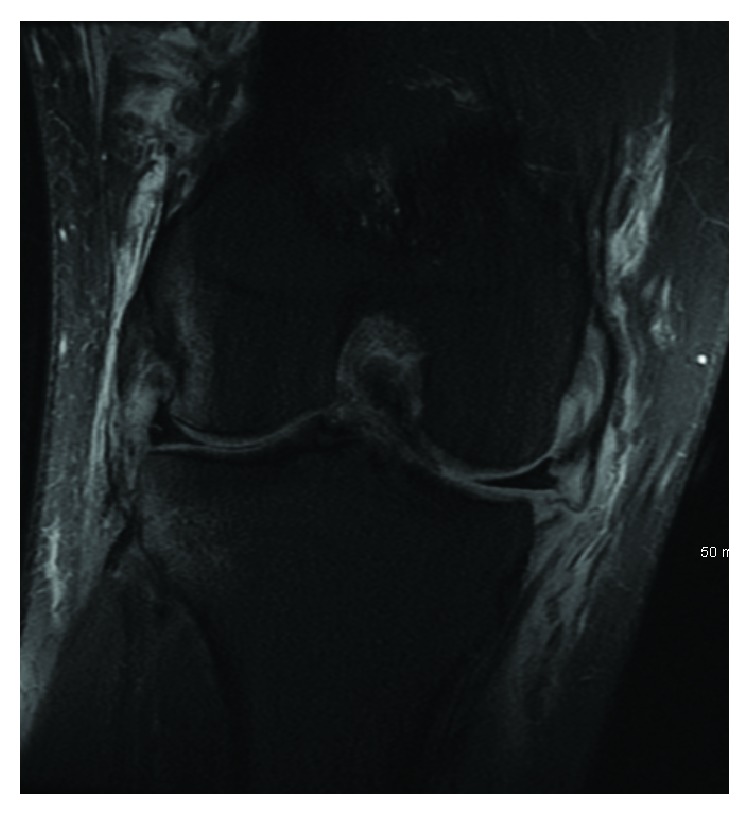
A T2 coronal MRI of the patient's right knee demonstrating a grade III MCL tear with partial extrusion of the medial meniscus.

**Figure 6:**
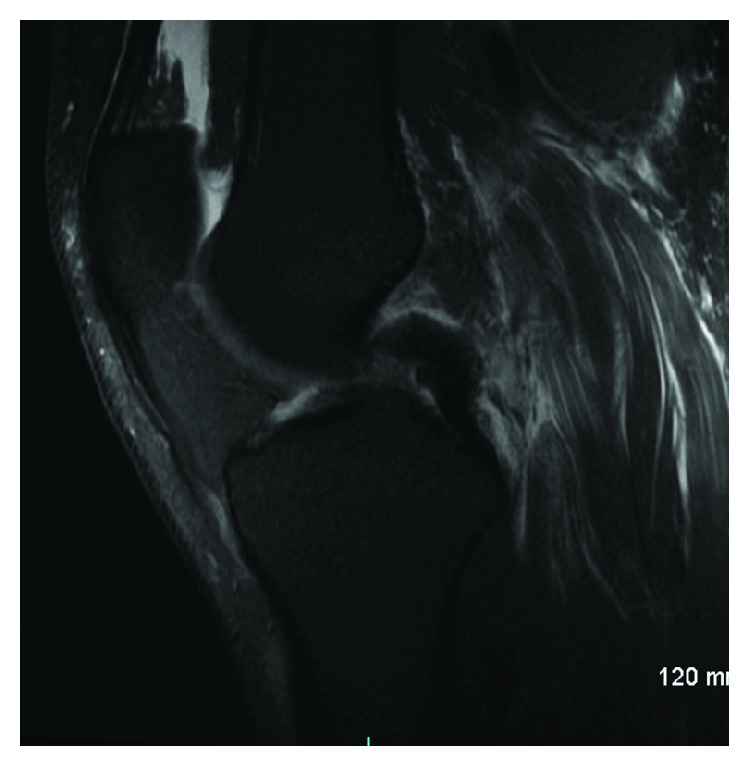
A T2 sagittal MRI of the patient's right knee demonstrating a grade II PCL tear.

Magnetic resonance imaging (MRI) of both knees was performed to evaluate for ligamentous injury. Left knee imaging demonstrated the Segond fracture along with a grade III lateral collateral ligament (LCL) tear with retraction (Figure 2), a grade II tear of the popliteus tendon and anterior cruciate ligament (ACL) (Figure 3), and a grade I medial collateral ligament (MCL) injury (Figure 4), as well as partial thickness tears of the biceps femoris and vastus medialis. Right knee imaging revealed a grade III tear of the ACL and MCL (Figure 5), grade II tears of the posterior cruciate ligament (PCL) (Figure 6), LCL, and popliteus tendon, and a medial meniscus tear. The patient was placed in bilateral hinged braces with the left knee unlocked and the right knee in locked extension to aid with transfers from a bed to a wheelchair. The patient was also given a left foot drop boot for a foot drop discovered during a secondary exam. On hospital day three, the patient was discharged home.

Nine days after the accident, the patient presented to the clinic. He noted that the left-sided foot drop was improving. On that side, he had 5/5 strength of his extensor hallucis longus and tibialis anterior (TA), without any sensory deficits in the peroneal nerve distributions. On the physical exam of the left knee, the Lachman maneuver was grade 2B (ACL injury with 5–10 mm translation without an endpoint), the varus stress test grade 3 (complete LCL tear with >10 mm opening of the lateral joint), and the valgus stress test grade 2 (MCL injury with 6–10 mm opening of the medial joint). The right lower extremity was also neurovascularly intact, and the right knee exam revealed a grade 2A Lachman maneuver (ACL injury with 5–10 mm translation and a firm endpoint), a grade 3 posterior drawer test (complete tear of PCL with >10 mm posterior tibial translation), and a grade 3 valgus stress test (MCL injury with 11–15 mm opening of the medial joint), with a presumptive positive dial maneuver on the right side at 30 and 90 degrees (consistent with PCL and posterolateral corner (PLC) injury). However, given that the patient had bilateral PLC injuries, this physical exam finding was somewhat subjective without a reference point on the contralateral side. Subtle gapping with varus stress was also documented.

Multiligamentous reconstructions of both knees were recommended (Table 1). The left knee was addressed first in order to explore and decompress the common peroneal nerve. In regard to the right knee, preoperative physical therapy was performed to restore range of motion (ROM) before undergoing surgery.

**Figure 7:**
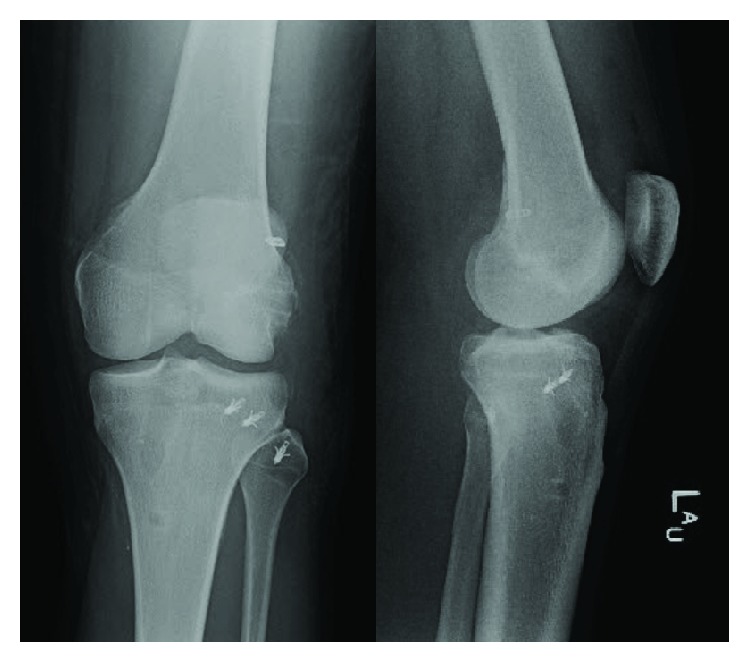
A postoperative anteroposterior and lateral radiograph of the patient's left knee following ACL reconstruction with a hamstring autograft augmented with an allograft, posterolateral corner reconstruction with an allograft, and repair of the native avulsed LCL and ALL with suture anchors.

Intraoperative findings of the left knee included a positive lateral gutter drive-through sign indicative of a PLC injury. The LCL was avulsed off the fibula, and the anterior lateral ligament (ALL) was also avulsed off the tibia. A greater than 50% disruption of the ACL was observed. Exam under anesthesia demonstrated a grade 2A Lachman maneuver, a grade 2 pivot shift, grade 3 varus instability, and instability on external rotation. The procedure included ACL reconstruction with a hamstring autograft augmented with an allograft, PLC reconstruction utilizing a TA allograft, and repair of the native avulsed LCL and ALL with suture anchors (Figure 7). First, the hamstrings were harvested and augmented with an allograft, and the tunnels for the ACL reconstruction were drilled. The PLC was then reconstructed using the anatomic technique described by Malanga et al. [12]. The native LCL was repaired using suture anchors with the overlying allograft reconstruction used to supplement it. The posterolateral capsule was then reefed into the LCL allograft reconstruction. Finally, the ACL graft was passed and fixed. Postoperatively, the patient was placed in a hinged brace locked in extension and was made toe-touch weight bearing.

**Figure 8:**
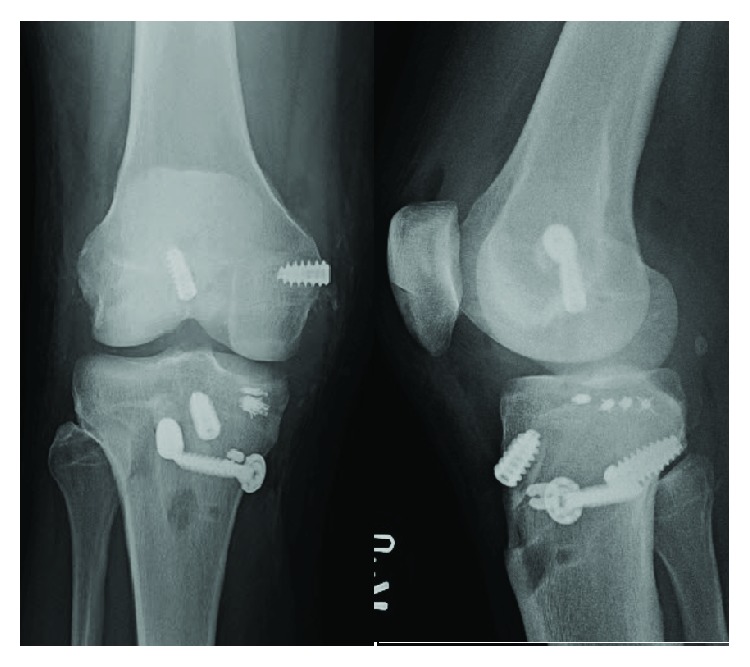
A postoperative anteroposterior and lateral radiograph of the patient's right knee following ACL reconstruction with a bone-tendon-bone autograft, PCL reconstruction with an allograft, MCL repair with additional allograft reconstruction, posterolateral corner reconstruction with an allograft, and repair of the posterior horn of the medial meniscus.

The decision was made to proceed with the right knee reconstruction one week later. Exam under anesthesia revealed a grade 2A Lachman maneuver, grade 3 posterior drawer test, a grade 3 varus stress test, and a grade 2 valgus stress test. Surgery included ACL reconstruction with a bone-tendon-bone (BTB) autograft, PCL reconstruction with an Achilles allograft, MCL primary repair with additional Achilles allograft reconstruction, PLC reconstruction with TA allograft, and repair of the posterior horn of the medial meniscus (Figure 8). The lateral exposure for the PLC reconstruction was performed first, and the blind-ended sockets and fibular tunnel were drilled. An open approach to the MCL was then performed, and the injured MCL was found and tagged for later repair. The BTB autograft was then harvested for the ACL reconstruction. At this point, the posterior horn of the medial meniscus was confirmed to be torn from its root so this was repaired using sutures passed through a tibial tunnel. Guide pins were then passed for the ACL and PCL tunnels to ensure there was no convergence. Both tunnels were then reamed and the grafts passed. The PCL was fixed first while the leg was flexed, and the ACL was then fixed with the leg in extension. The MCL repair and reconstruction with an allograft were completed using the surgical technique described by Sekiya et al. [13], followed by the PLC reconstruction which was carried out utilizing the same method noted previously. Postoperative immobilization and weight bearing status were the same as those in the contralateral side.

Range of motion and physical therapy rehabilitation began at 1 week postoperatively. Early exercises included isometric activities to strengthen the quadriceps and patella mobilization exercises. Both knees were kept in a brace locked in extension with minimal weight bearing the first six weeks following surgery. Six weeks after the initial reconstruction, the patient was instructed to begin weight bearing with crutch assistance, starting in extension and then unlocking the straight leg brace to 90° of flexion. At eight weeks post-op, the patient was transitioned out of knee braces and then given clearance to return to work ten weeks after surgery.

**Figure 9:**
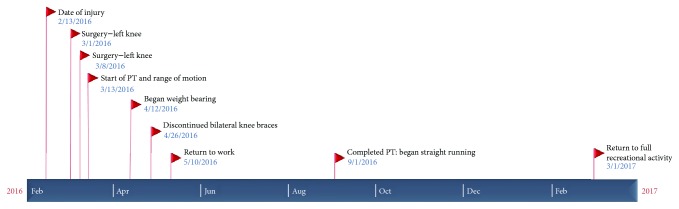
Timeline of the patient's surgical procedures and rehabilitation course.

At six months post-op, the patient completed physical therapy. On the physical exam, the patient's knees demonstrated full ROM bilaterally with a grade 1A Lachman maneuver in both knees. The right knee also had a grade 2A posterior drawer test without sag. At that time, clearance was given to begin straight running. At one year post-op, the patient had returned to jogging and playing basketball recreationally and was able to participate in strenuous work (Table 2, Figure 9).

## 3. Discussion

Bilateral MLI occur as a result of high-energy mechanisms, with most reported cases resulting from motor vehicle accidents or motorcycle accidents [8–11]. Bilateral injuries are rare occurring in only 4-5% of all patients who sustain MLI. Compared to patients sustaining unilateral MLI, patients with bilateral injuries have significantly higher Injury Severity Scores, as well as more frequent chest, abdominal, and single-level spine injuries [11]. Given the higher incidence of concomitant injuries with these patients, careful evaluation in conjunction with a trauma team is crucial on presentation to assess for life- and limb-threatening injuries [5].

A thorough history and physical exam are necessary following knee dislocation to assess for MLI. In the acute setting, the examination can be severely limited due to patients' apprehension, guarding, and swelling [6]. Additionally, in the setting of bilateral MLI, the examiner has no contralateral reference point to compare the injured knee to when assessing for instability. Despite these challenges, a physical exam is imperative to assess the extent of ligamentous injuries following knee dislocation. Additionally, radiographs and advanced imaging should also be obtained to evaluate for the presence of periarticular fractures, tibial plateau fractures, and tendinous avulsion fractures [14]. MRI has been found to be highly sensitive to diagnosing meniscal, cruciate, and collateral ligament injuries of the knee [15].

The anterior drawer test and Lachman maneuver can both be used to detect and evaluate the extent of ACL tears, with the Lachman maneuver demonstrating higher sensitivity and specificity over the anterior drawer test [12]. In patients with MLI and concurrent PCL tears, posterior subluxation of the tibia can obscure findings from these exams [6]. Additionally, the pivot shift test used to assess for anterior knee instability loses some utility with MLI of the knee because of the inability to control for hip and leg position.

The posterior drawer test and posterior sag test are used to assess for PCL tears. In the case of a grade 3 posterior drawer test (>10 mm of posterior translation), a concomitant PCL and PLC injury should be suspected [13]. PLC stability is evaluated using the dial test. The knee is positioned at 30° and then 90° of flexion with external rotation applied to the foot. The evaluator then measures the amount of external rotation of the knee with ≥10° difference deemed significant. An isolated PLC injury is suspected with increased external rotation at 30° alone while a concomitant PCL and PLC injury is suspected with increased external rotation at both 30° and 90°. An isolated PCL injury is present with increased external rotation at 90° only [16]. This maneuver relies on contralateral comparison to determine significant differences, which, in the case of our patient with suspected bilateral PLC injuries, limited its utility. Furthermore, the dial test can also be positive in cases of isolated or combined medial-sided injuries. Therefore, it is important to concurrently examine a patient for the degree of anteromedial or posterolateral tibial rotation to distinguish PLC versus posteromedial injury of the knee [17].

Valgus and varus stress tests are used to evaluate the MCL and LCL, respectively. Increased medial joint opening with valgus stress while the knee is in full extension suggests concomitant cruciate and/or posteromedial capsular injury. Excessive lateral joint opening with varus stress suggests concomitant PLC and/or cruciate ligament injury [18]. Furthermore, varus and valgus stress radiographs provide useful adjuncts to the physical examination as they can be used to further evaluate the extent of these injuries preoperatively.

Careful assessment for a vascular injury is critical as failure to recognize such an injury may result in loss of the limb if not addressed emergently. A recent systematic review of 862 patients who experienced knee dislocations in the literature demonstrated a weighted frequency of 18% who sustained vascular injuries [19]. Conversely, a larger study of 8050 limbs with knee dislocations identified from a large private-payer database demonstrated 267 concomitant vascular injuries for an overall frequency of 3.3% [20]. While routine arteriography was previously the standard of care [19], more recent recommendations suggest selective arteriograms for cases in which an ankle-brachial index < 0.8 is observed with a well-perfused foot, with any changes in color and temperature, or with diminished pulsations in the ipsilateral foot, or for the case of an expanding hematoma. If the ABI is normal (≥0.9), no further testing is necessary, but serial exams should be performed to closely monitor the vascular status of the affected extremity.

Early identification of nerve injuries will help guide management of these injuries and potentially prevent permanent damage. In a retrospective study performed investigating MLI patterns at a level I trauma center, the incidence of peroneal nerve injury was 25% and highly associated with PLC injuries [5]. The mechanism of injury typically involves traction on the peroneal nerve resulting from a substantial varus force applied to the knee [21]. In the case of incomplete nerve palsy, the majority of patients will make a complete recovery of nerve function. Surgical intervention is indicated for all patients with complete palsies [21].

### 3.1. Surgical Management

Previous literature has demonstrated superior outcomes in surgically treated patients compared to those managed nonoperatively [7, 22–24]. In a recent review, operatively managed knee dislocations had superior functional outcomes with lower rates of contracture and instability and increased return to preinjury levels of activity [23]. In patients with significant comorbidities and severe concomitant injuries or those with limited functional status, nonoperative treatment may be considered. Due to this patient's young age and preinjury activity level, he was felt to be an ideal surgical candidate.

Timing of multiligamentous reconstruction has been an area of ongoing debate. Acute reconstructions refer to those that occur within 2-3 weeks of injury, while delayed reconstructions are those performed after that time [7, 11, 22]. There has been an increasing consensus that acute interventions produce superior subjective and objective functional outcomes as well as improved ligamentous stability [22, 25–29]. A systematic review by Mook et al. demonstrated that acute reconstructions are associated with significantly higher odds of residual anterior knee instability, flexion deficits, and the need for additional surgeries for manipulation or arthrolysis [30]. Arguments for delayed reconstruction include the opportunity to increase ROM of the injured knee prior to surgery as well as to allow other injuries in extra-articular structures and soft tissue to have increased time to heal, potentially avoiding further operative interventions [22, 31].

### 3.2. Operative Technique and Literature Review with a Focus on Controversies

Reconstructive techniques have shown improved results and decreased failure rates compared to primary repairs of injuries to the MCL, posteromedial corner, and PLC [32, 33]. Often, reconstruction of cruciate ligaments of the knee is augmented with allografts or autografts due to decreased failure rates and residual laxity compared to earlier reconstructive/repair techniques [32–35].

#### 3.2.1. ACL

Due to lack of studies comparing different ACL reconstruction techniques in the setting of the MLI, surgical techniques are typically dictated by a surgeon's preference [36]. The current patient underwent ACL reconstruction with a hamstring autograft of the left knee, augmented with an allograft. The hamstring autograft was favored because it has shown to have less donor site morbidity and pain when compared to the bone-tendon-bone autograft [37–39]. Augmentation of the hamstring autograft was performed because the native autograft has a diameter of <8 mm, which has been shown to portend failure [40–42]. The right knee underwent ACL reconstruction with a bone-tendon-bone autograft. A hamstring autograft was not preferred on the right knee given concomitant MCL injury and the role of hamstring tendons in dynamic stabilization of the medial knee.

#### 3.2.2. PCL

No graft or surgical technique as the gold standard for PCL reconstructions in MLI exists. The leading technique options include tibial inlay and transtibial reconstructions [36, 43]. Many grafts have been utilized including the Achilles allograft or hamstring and patellar tendon autograft [36, 43]. Right knee PCL reconstruction utilizing an Achilles allograft was the preferred method in our patient.

#### 3.2.3. MCL and PMC

Depending on the severity of injury to medial-sided structures of the knee, both repair and reconstruction may be considered. Avulsion of the MCL can often be repaired using suture anchors for reattachment, while midsubstance damage will typically require reconstruction with or without graft augmentation [32]. Irrespective of the technique used, anatomic and isometric arrangement is crucial and should be tested arthroscopically during range of motion manipulation [43, 44]. Newer techniques promote the use of an Achilles allograft or a modified Bosworth technique using a semitendinosus graft [44, 45]. Our patient's right knee underwent primary repair of the MCL with an Achilles allograft as well as medial meniscus repair.

#### 3.2.4. LCL and PLC

Techniques to address PLC injuries include both primary repair and reconstruction. Repair should be considered with osseous injuries such as an arcuate complex avulsion [36, 43]. The preferred reconstructive technique is an anatomic approach to restore native anatomy [43, 46–48]. Isolation of the peroneal nerve for protection is imperative, regardless of the technique utilized. Autograft tissues including those of semitendinosus tendon, biceps tendon, and split biceps tendon are used. Allograft tissues including those of TA, Achilles tendon, and bone-patellar tendon bone can also be used [36]. In our patient, the left knee underwent PLC reconstruction utilizing a semitendinosus allograft with repair of the native LCL and repair of the ALL with suture anchors, while the right knee underwent PLC repair with a TA allograft.

### 3.3. Complications and Comorbidities

Postsurgical complications occur at a much higher incidence in MLI as compared to single-cruciate-ligament injuries [49–52]. Some studies have suggested a direct correlation between the increased number of injured ligaments and obesity with the overall rate of complications [2, 49, 53]. Common complications that studies have addressed include high postoperative infection rates, arthrofibrosis, residual laxity, failure rates, and posttraumatic osteoarthritis. Infection rates can range anywhere from 0% to 17.4% in MLI [2, 49]. Arthrofibrosis is more common after severe injuries, acute reconstruction, and medial-sided injury repair [7, 22, 30, 36, 44, 45, 49]. Posttraumatic osteoarthritis can emerge in up to 53% of knees, due to cartilage injury and residual instability postoperatively [36, 43].

## 4. Conclusion

Bilateral knee dislocations are rare, and literature detailing the treatment of these types of injuries is largely limited to unilateral knee injuries. We detailed the perioperative management and operative techniques used to treat a patient with bilateral MLI who went on to regain excellent function one year postoperatively. This case highlights that each MLI represents a unique challenge to the treating surgeon regarding timing, sequence of reconstruction, and postoperative rehabilitation protocol.

## Figures and Tables

**Table 1:** Summary of knee injuries and surgical treatment.

	Left knee	Right knee
*Injuries sustained*		
ACL	Grade 2B	Grade 2A
PCL	Grade 2	Grade 3
MCL	Grade 2	Grade 3
LCL	Grade 3	Grade 2
*Nerve injury*	Common peroneal nerve	—
*Time until surgery*	2 weeks	3 weeks
*Considerations*	Decompression of the common peroneal nerve	Pre-op PT to improve ROM
*Surgical treatment*		
ACL	Hamstring autograft with allograft augmentation	Bone-tendon-bone autograft
PCL	N/A	Achilles allograft
MCL	N/A	Repair with Achilles allograft reconstruction
LCL	Repair with suture anchors	N/A
PLC	Reconstruction with a tibialis anterior allograft	Reconstruction with a tibialis anterior allograft
ALL	Repair with suture anchors	N/A
Medial meniscus	N/A	Posterior horn repair

**Table 2:** Patient-reported functional outcomes of the left and right knee preoperatively compared to 12 months postoperatively using International Knee Documentation Committee (IKDC) and Knee Injury and Osteoarthritis Outcome Score (KOOS) patient-reported outcome measures.

	Pre-op	12-month follow-up
*IKDC*		
L knee	36.8	87.4
R knee	19.5	81.6
*KOOS*		
L knee	42.3	98.8
R knee	55.4	89.9
